# Testing, Training, and Optimising Performance of Track Cyclists: A Systematic Mapping Review

**DOI:** 10.1007/s40279-021-01565-z

**Published:** 2021-09-30

**Authors:** Antony M. J. Stadnyk, Franco M. Impellizzeri, Jamie Stanley, Paolo Menaspà, Katie M. Slattery

**Affiliations:** 1 School of Sport, Exercise, and Rehabilitation, University of Technology Sydney, Sydney, NSW Australia; 2 New South Wales Institute of Sport, Sydney, NSW Australia; 3 South Australian Sports Institute, Adelaide, SA Australia; 4 Australian Cycling Team, Adelaide, SA Australia; 5 Allied Health and Human Performance, University of South Australia, Adelaide, SA Australia; 6 Centre for Exercise and Sports Science Research, School of Medical and Health Sciences, Edith Cowan University, Joondalup, WA Australia

## Abstract

**Background:**

Track cyclists must develop mental, physical, tactical and technical capabilities to achieve success at an elite level. Given the importance of these components in determining performance, it is of interest to understand the volume of evidence to support implementation in practice by coaches, practitioners, and athletes.

**Objective:**

The aim of this study was to conduct a systematic mapping review to describe the current scale and density of research for testing, training and optimising performance in track cycling.

**Methods:**

All publications involving track cyclist participants were reviewed from four databases (PubMed, SPORTDiscus, Academic Search Complete, Cochrane Library) plus additional sources. Search results returned 4019 records, of which 71 met the inclusion criteria for the review.

**Results:**

The review revealed most published track cycling research investigated athlete testing followed by performance optimisation, with training being the least addressed domain. Research on the physical components of track cycling has been published far more frequently than for tactical or technical components, and only one study was published on the mental components of track cycling. No true experimental research using track cyclists has been published, with 51 non-experimental and 20 quasi-experimental study designs.

**Conclusions:**

Research in track cycling has been growing steadily. However, it is evident there is a clear preference toward understanding the physical—rather than mental, tactical, or technical—demands of track cycling. Future research should investigate how this aligns with coach, practitioner, and athlete needs for achieving track cycling success.

**Registration:**

This systematic mapping review was registered on the Open Science Framework (osf.io/wt7eq).

**Supplementary Information:**

The online version contains supplementary material available at 10.1007/s40279-021-01565-z.

## Key Points

**Table:** 

Relative to other cycling disciplines, there is limited research to support evidence-based practices for developing track cycling, and while interest has grown significantly in the past decade, no true experimental studies have been conducted to date, therefore our limited understanding of causal pathways between interventions and performance persists.
The physical component of performance is the most commonly researched, across a broad range of topics, while the mental component is almost completely unaddressed. Testing is the most commonly researched domain of athlete preparation, whereas training is the least investigated.
Future research should seek to understand the importance of each component of performance within the domains of athlete preparation in track cycling, and how the current evidence aligns with, and could better address, the needs of coaches and practitioners implementing research in practice to develop track cyclists.

## Introduction

Track cycling, at an international elite level, is a sport that requires athletes to ride around a 250 m banked track on fixed-gear bicycles with the primary objective of finishing a race as quickly as possible. Track cycling races are generally categorised as sprint (e.g., team sprint, keirin) or endurance (e.g., team pursuit, bunch races), depending on their distance and demands. Sprint events can last as little as 9 s, whereas endurance events are over 3.5 min for pursuits, and longer for bunch races (typically 10–60 min duration). Track cycling is a demanding sport [1] and developing athletes for competition at an elite level can be a circuitous challenge. To make the process as efficient and effective as possible, coaches, practitioners (e.g., physiologists, strength and conditioning coaches, biomechanists, engineers) and the athletes themselves seek evidence that will help them to reach training and performance goals. Under the evidence-based practice framework, this relies on the best-available scientific evidence supplementing our experiences and values to inform our methods [2]. Many world records within track cycling have been broken and rebroken in recent years through both human- and equipment-driven performance improvement. Such performances may indicate that the demands of track cycling events are changing; as performance times shorten, relative metabolic contributions may be altered [3], along with the physiological and morphological profiles of the athletes best-suited to the various races. However, it is unclear if the available scientific evidence is relevant or is keeping up to date so that true evidence-based practice can be feasibly achieved.

Despite being the cycling discipline offering the highest number of Olympic and Paralympic medals, track cycling lacks the breadth or depth of scientific evidence that is comparatively available for road cycling and mountain biking. While some fundamentals of the sport are similar to those close relatives (e.g., physiological profiles [4]), there are also distinctions (e.g., fixed-gear cycling, tactical and technical demands) that can be made that require a greater specificity, and subsequent application, of knowledge to achieve desired results. The ‘adopting’ and ‘adapting’ of evidence from one sporting discipline to another, while potentially useful in the short term, may be an imperfect solution that, in the long term, can lead to imperfect results and missed opportunities for those implementing the findings in practice.

While groups have characterised the demands of cycling broadly across all disciplines [4], various athletes’ training profiles [5], and studied physiological interventions [6], none have reviewed all available literature on track cycling to evaluate the current quantity or quality of evidence available. As such, a systematic mapping review was completed. This style of review follows standard systematic search principles, then categorises each study by a subset of characteristics of value to researchers and practitioners alike [7, 8]. Mapping reviews identify gaps in the literature and a path toward necessary further research or reviews. The data are presented in a user-friendly manner to aid the reader in visualising the scale and density of a research area [8]. The present review, along with forthcoming research by the authors, will inform the future development of a framework for researchers and practitioners to guide research and the development of track cycling athletes.

## Methods

### Research Question

We conducted a systematic mapping review to answer the following research questions:

1. What is the quantity and type of research published on track cycling, and what temporal trends, if any, exist?
2. Which domains of athlete preparation have been the focus of track cycling research?
3. Which components of athlete performance has track cycling research investigated?

### Search Strategy and Screening Process

**Fig. 1:**
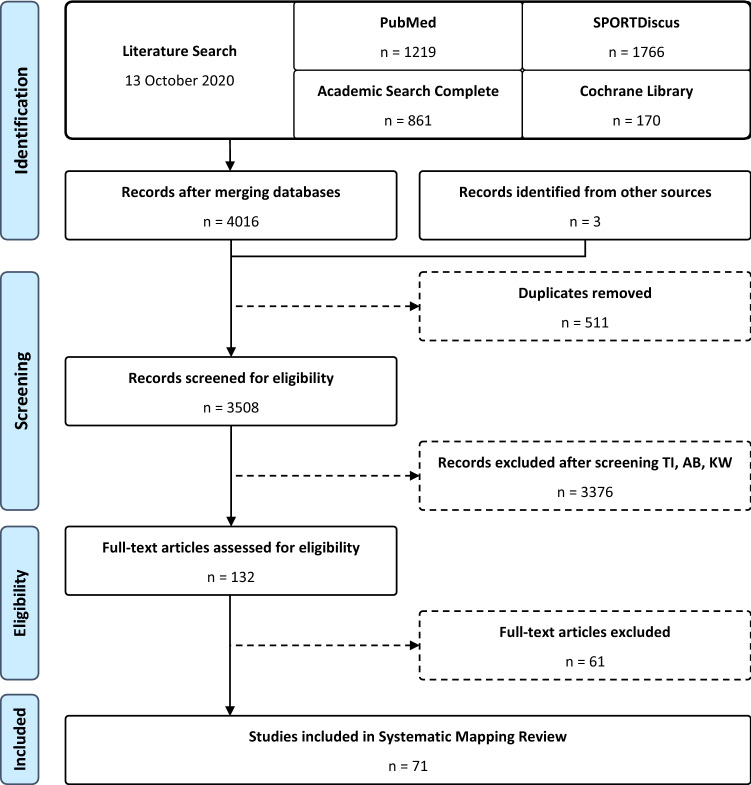
PRISMA flowchart of the literature search and screening process. *PRISMA* Preferred Reporting Items for Systematic Reviews and Meta-Analyses, *TI* title, *AB* abstract, *KW* keywords

We conducted a systematic mapping review of literature involving track cyclists using the Academic Search Complete, Cochrane Library, PubMed, and SPORTDiscus databases. A search strategy was developed to identify all relevant studies using an extensive list of terms related to track cycling. Scoping searches were conducted on each database using variations of search terms, operators, and wildcards to maximise the number of search results returned with the final search strategy. Due to wildcards having an undesirable effect on search results within and between databases (e.g., omitting potentially relevant search results), a standardised search strategy that included both singular and pluralised variations of search terms was chosen. All databases were first searched from the earliest record up to and including 28 August 2019. The search was updated with new results from all databases on 13 October 2020. Potentially relevant literature not found in database searches were identified from other sources (e.g., included studies’ reference lists) and were included for screening. The literature search and screening process is outlined in the Preferred Reporting Items for Systematic Reviews and Meta-Analyses (PRISMA) diagram [9] shown in Fig. 1. The review protocol was registered with Open Science Framework (OSF; ID: wt7eq), an open public data repository.

All records were uploaded to Covidence (Veritas Health Information, Melbourne, VIC, Australia), a web-based screening tool, and were initially screened by title and abstract against the selection criteria (see below). If an article did not clearly meet the selection criteria in the initial screening process, it was included in the full-text screening phase. Articles were initially screened by AS and KS, with articles approved by both reviewers being sent to the following stage, while articles rejected by both were noted before being removed. If there was disagreement between reviewers, the article was sent to a third reviewer (FMI) to approve or reject. This process was performed during the screening of abstracts and, subsequently, full-text articles. The inter-rater agreement Cohen’s *κ* was 0.62 (97.5%) and 0.70 (84.7%) for the title/abstract and full-text screening phases, respectively. A reason for exclusion was noted for each article removed during the full-text article screening stage. Best efforts were made by the researchers to find all full-text articles; however, in instances where these were not found (*n* = 13; 9.8%), researchers screened the available information (e.g., abstracts) and, if sufficient detail was present, they were included for analysis. As such, articles without full-text available were not necessarily excluded, which is a benefit of mapping reviews.

### Inclusion and Exclusion Criteria

Our inclusion criteria were studies that (1) specifically involved competitive or trained track cyclists; (2) described methods employed for testing, training, and/or optimising the performance of track cyclists, or described the characteristics of testing, training, and/or performance of track cyclists; and (3) were original research (i.e., not reviews, book chapters, opinions, editorials). Exclusion criteria included studies that (1) did not delineate results if the sample included non-track cyclists; and (2) were primarily nutritional interventions. As this was a mapping review, no quality appraisal was performed and all studies meeting our criteria were included [7].

### Data Extraction

To create a map of existing track cycling literature, AS extracted all data pertaining to study details (experimental design, duration, country), population (sample size, age, training status, anthropometry, athlete country), specific measures/methods/interventions investigated, and general outcomes reported. Extracted data were entered into a custom-made online spreadsheet allowing for simultaneous data entry and review by multiple authors. After initial data extraction, a random sample of studies was allocated to each of the remaining authors to cross-check extracted data against the respective full-text article to ensure accuracy. As mapping reviews do not necessarily synthesise all extracted data [8], a tabular summary of the data has not been provided within this text; however, select data pertinent to the research aims and questions have been reported throughout the following sections. The complete extracted data file is available to view online (osf.io/wt7eq).

### Categorisation of Studies

**Fig. 2:**
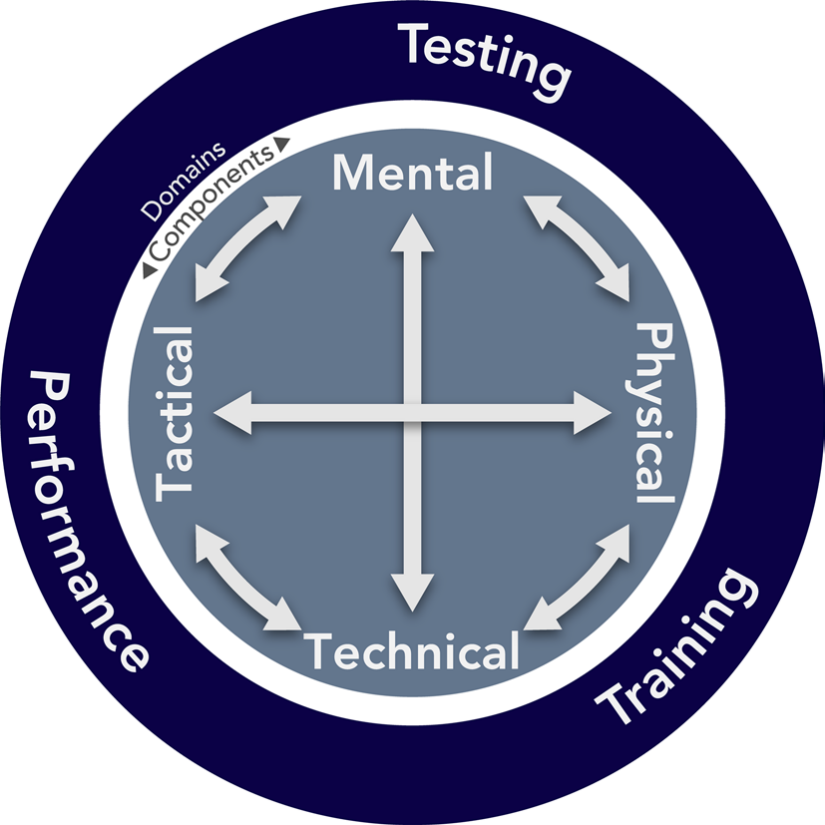
A visual representation of the interface between the distinct, but interrelated, Domains of Athlete Preparation (outer circle), and Components of Athlete Performance (inner circle); arrows indicate potential interactions between the components, which can be addressed within each of the domains

The primary feature of evidence mapping is the categorisation of studies by generalised characteristics. As is common in qualitative research, evidence mapping involves a form of thematic analysis to identify and examine emergent themes or patterns within data. The primary categorisations related to the distinct, but interrelated, domains or phases of the ‘circular’ process of athlete preparation—testing, training, and performance optimisation (Fig. 2). In this process, athletes are tested, test results inform training prescription, and training influences capacity to perform, which itself can be optimised. The performance or repeated testing restarts this process. For the purposes of this mapping review, we constructed operational definitions for each of these domains/phases to guide categorisation now and in any future research. These definitions are:

- *Testing* Describes the characteristics of track cyclists or quantifies a component of track cycling performance for the purpose of establishing normative test values to make comparisons within and between athletes; or, assesses a specific, isolated/acute test’s validity, reliability, or relevance to a component of track cycling performance, either independently or in comparison with another testing method.
- *Training* Assesses training practices or methods prescribed to track cyclists for the purpose of establishing inter- and/or intraindividual acute or chronic responses and/or their effect on a component of track cycling performance, and strategies that promote optimal adaptation.
- *Performance Optimisation* Describes or assesses practices related to understanding or improving execution of track cycling performance; may be practical or theoretical in nature, including, but not limited to, tactics, skills and technique, athlete–equipment interaction, strategies to optimise performance.

Sports performance is the product of a complex interplay between mental, physical, tactical, and technical components [10–12], therefore these were used as secondary categorisations to highlight how frequently these areas have been addressed in research. For the purposes of this review, studies were classified as per the following descriptors:

- *Mental* Psychological constructs within track cycling and cyclists.
- *Physical* Physiological capacities or demands of track cyclists/cycling.
- *Tactical* Strategic considerations within track cycling, e.g., pacing, interactions with other individuals such as in team pursuit or match sprints.
- *Technical* Biomechanics, aerodynamics, acquisition and execution of individual riding skills.

All studies were initially categorised by AS and KS. Differences in categorisation were discussed and resolved by these two authors plus FMI. Studies were categorised in all applicable domains and components.

## Results

### Growing Research Interest in Track Cycling

**Fig. 3:**
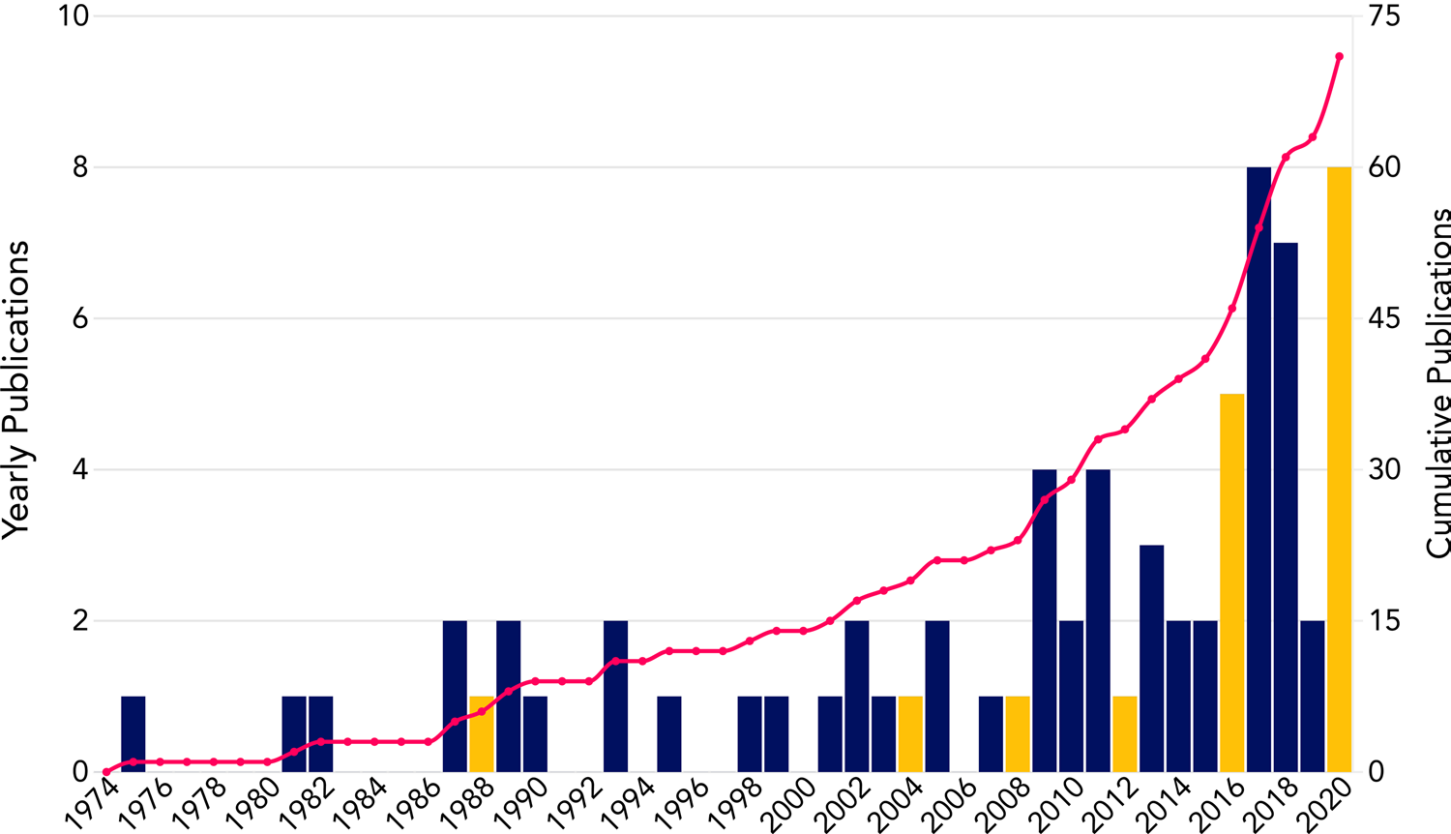
Number of track cycling publications by year; cumulative number over time is represented by the red line, and Olympic Games years are coloured gold (note: Tokyo 2020 Olympic Games were postponed to 2021)

The number of publications per year in track cycling has steadily risen. A sharp increase in quantity from 2009 onwards is evident, and there were as many studies published between 2012 and 2020 as there were from 1975 to 2012. Whether by coincidence or other underlying reason, there appears to be a trend for a small spike in publications in the period immediately after the Olympic Games (coloured gold in Fig. 3). The median number of publications per year peaks at 2 (interquartile range [IQR] 0.5–2.5) in the year immediately following the Olympic Games and declines gradually at two [1 (0–2)] and three [1 (0.75–2)] years post Olympics. A median of 0.5 (0–1) studies were published per Olympic Games year.

Eighteen nations, per first author affiliation (20 nations in total), have contributed to the track cycling literature, with global interest increasing across each decade. Between 1975 and 1999, only seven countries (Australia, Canada, Czechoslovakia, France, Japan, UK, USA) had published track cycling research, with research groups from Australia conducting half of those studies. Each subsequent decade has seen the added contributions of a further four countries (Brazil, China, Germany, Spain) between 2000 and 2009, seven countries (Austria, Denmark, The Netherlands, New Zealand, Norway, Poland, Switzerland) between 2010 and 2019, and two countries (India, South Africa) in 2020. Australian institution-affiliated researchers have had the largest contribution with 24 publications and is the only country to have made contributions in each decade since 1980. The UK follow with 18 publications and, along with France- and USA-based researchers, have made contributions in four of the decades since 1975.

### Research Design

Each study was assessed for their research design as per the descriptors provided by Page [13]. The scientific subdisciplines of each study, organised by research method and type, are shown in Table 1. As is common across elite sport and the sport science literature, there is a lack of true experimental research, i.e., randomised control trials [RCTs] published on track cyclists. Of the 71 publications that met our inclusion criteria, none had a true experimental study design. Over 70% of the included studies (*n* = 51) were non-experimental study designs, with the remainder (28.2%) being quasi-experimental.

**Table 1:** Research designs used by included studies (reference numbers in brackets), categorised by their respective scientific subdisciplines; the full dataset with category filters is available at osf.io/wt7eq

	Non-experimental		Experimental
	Descriptive	Exploratory	Quasi
Aerodynamics	[14]		
+ Biomechanics			[15]
+ Tactics/Strategy	[16]		
Anthropometry	[17, 18]		[19, 20]
+ Aerodynamics	[21]		
Biomechanics	[22]	[23]	[24, 25]
+ Physiology		[26]	
Physiology	[27–34]	[35–46]	[6, 47–60]
+ Aerodynamics	[61]		
+ Anthropometry	[5]	[62–67]	
+ Biomechanics	[68]		
Psychology		[69]	
Skill acquisition/technique			[70]
Tactics/strategy	[71–74]	[75–77]	[78]
+ Aerodynamics	[79]		
+ Physiology	[80]	[81]	
+ Skill Acquisition		[82]	

Descriptive studies (e.g., case-study, observational) were the most common (*n* = 23) non-experimental research type, followed by exploratory (e.g., cohort, correlational; *n* = 28). Of the quasi-experimental studies, 14 used a pre-post or repeated measures design, while six used a crossover design. Studies ranged in duration from single days or testing sessions (*n* = 19) to 1 year or longer (*n* = 11). The median study duration was 4 days, with 16 studies of 2–4 days’ duration and 11 studies of 10–28 days’ duration. There were six studies between 3 and 6 months’ duration. The median duration of quasi-experimental studies was 15 days, versus 3 days for non-experimental studies. All of the studies that lasted a year or longer were non-experimental.

Only two of the studies investigated solely female track cyclists [16, 17], whereas 70.4% (*n* = 50) of studies assessed only male track cyclists. The remainder were mixed-sex samples (*n* = 19; 26.8%). There was a similar mix of studies involving track sprint (*n* = 29) and track endurance (*n* = 30) cyclists. The remaining 12 studies had a mixed sample of the two categories of riders, many of which delineated results to distinguish the differing demands of their respective events. Most studies involved a heterogeneous mix of track cyclists with varying training statuses and competing at levels ranging from ‘Local/Regional’ (*n* = 7) to ‘Olympic’ (*n* = 31) level. There were seven studies involving athletes competing at a State level, and 26 studies sampled ‘National’-level track cyclists. Over 60% (*n* = 43) of the included studies involved athletes competing at or above ‘International’ level, e.g., World Championships, World Cups. In four of the studies, it was unclear what the training status of the track cyclists was. Athletes’ ages ranged from teen/adolescent to Masters grade (i.e., over 35 years of age), although the majority of participants were adult/senior (i.e., > 18 years of age competition level).

### Domains of Athlete Preparation

**Fig. 4:**
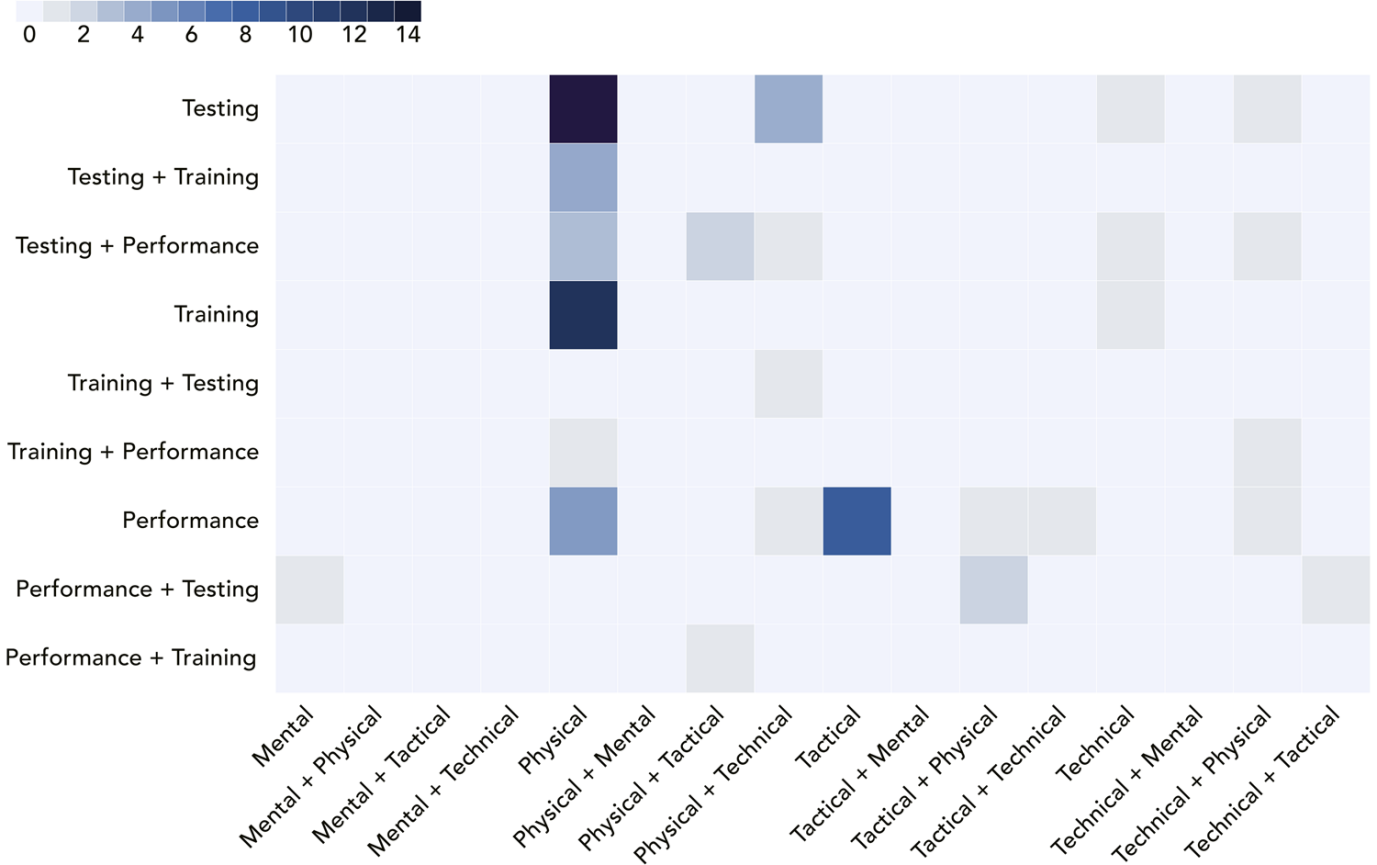
Heat map of the frequency of studies as categorised within the Domains of Athlete Preparation (*y*-axis) and Components of Athlete Performance (*x*-axis); colours correspond to the number of articles, with darker squares indicating higher research density (key, top left)

Athlete preparation for competition at an elite level is a process of Testing, followed by Training, followed by (optimising) Performance, repeated in a circular fashion with the aim of continual development and improvement. Of these three domains, or phases, of preparation in track cycling, Testing has received the most attention and Training the least (Fig. 4). Testing within track cycling was investigated in 38 (53.5%) papers, and was the primary domain of interest in 33 (46.5%) studies. Training was the primary domain of interest in 16 (22.5%) studies, and a secondary domain in a further 5 (7.0%) studies. Performance was the primary focus of investigation in 22 (31.0%) studies, and 10 other studies (14.1%) included an aspect of Performance as a secondary focus. Among the studies that had both a primary and secondary domain of focus (*n* = 20; 28.2%), the most common pairings were Testing and Performance (*n* = 12; 16.9%), followed by Testing and Training (*n* = 5; 7.0%), and Training and Performance (*n* = 3; 4.2%).

### Components of Athlete Performance

Athletes must develop a range of capabilities across four key components of performance, which they must then integrate during competition in order to put themselves in a position for success [10–12]. Understanding each of those components (and their composite elements) and how they interact is imperative for developing a well-rounded athlete and consistent and optimal performances. The *Physical* component of sporting performance was the most frequent focus of the included studies in this review across various scientific subdisciplines, including physiology and anthropometry. Conversely, the *Mental* component was the least examined, with only a single study published on pre-competition stress [69]. Across all included studies, the *Physical* component was the primary focus in 50 (70.4%) studies, and as a secondary focus in a further seven (9.9%) papers. *Tactical* (e.g., tactics and strategy) and *Technical* (e.g., aerodynamics and technique) components of performance were investigated in 16 (22.5%) articles each and were the primary focus in 12 (16.9%) and 8 (11.3%) of those studies, respectively. Of the 18 (25.4%) studies that had both a primary and secondary component, the most frequent interactions were between *Physical* and *Technical *(*n* = 11; 15.5%), *Physical* and *Tactical* (*n* = 6; 8.5%), and *Tactical* and *Technical* (*n* = 2; 2.8%).

## Discussion

### Summary of Findings

This is the first systematic mapping review of the track cycling literature. The study included all papers published until October 2020. The results provide a snapshot of the published knowledge existing in the sport of track cycling and provide the reader an understanding of the domains and components that have, to date, received the most and least attention. The review also provides an overview of the research designs that have been employed, which may provide a proxy of the quality of evidence upon which testing, training, and/or performance decisions can be made in practice.

Unsurprisingly, it is evident that the *Physical* components of athlete performance are the most investigated within the track cycling literature, reflecting its critical importance [1]. The studies included investigations of test protocols, e.g., critical power and W’ [35, 36, 66]; establishing indices/requirements of performance [5, 61, 62]; normative values for body composition [17, 20] and post-competition lactate accumulation [27]; and the efficacy of warm-up protocols [6, 59] and recovery modalities [52, 55, 56]. However, there is clearly less published research on the tactical and technical components despite their importance in various sprint (e.g., match sprint, keirin) and endurance (e.g., team pursuit, bunch) events [73, 75, 76]. In the case of mental components of performance, which have only been addressed in one study, the broader sport psychology literature may be reasonably applicable within track cycling. However, it may still be useful to develop a profile of the mental skills and strategies employed by track cyclists to meet the psychological demands of competition. Areas that may be of interest in track cycling that have been studied in road cycling or other sports include mental preparation for multiple races (e.g., qualifying-to-finals, timed-to-tactical races, omnium); improving cognitive resilience for decision making (e.g., in bunch racing or match sprints); developing leadership skills that can contribute to social labouring (e.g., in team pursuit).

The difficulties of conducting RCTs in elite sport are well known [2] due to the small samples of homogenous athletes and the impracticality of manipulating training in the randomisation of athletes to different interventions with potentially detrimental impacts on performance. This fact is reflected in the lack of true experimental research designs. While several studies included within this review have utilised non- and quasi-experimental research designs to examine various training interventions [e.g., 30, 50, 53, 57] and have shown promising results, the inevitable risk of bias demands greater scrutiny and caution prior to implementation in practice.

The observed trend of declining publications with increasing proximity to the Olympic Games start date can be attributed to a number of possible factors. For track cycling research and performance programmes, there may be reduced time availability to publish as a result of a shift in resource allocation toward athlete preparation. Alternatively, it may point to an element of secrecy among competitors. Track cycling, as with other Olympic sports, is highly competitive, therefore it is possible that researchers deliberately withhold research from publication until after the pinnacle event as a means of limiting valuable findings and information being utilised by competing nations. Due to the low number of annual publications both prior to 2009 and generally, it is difficult to conclude this correlation is the result of anything more than chance, but it is nonetheless a notable and interesting trend.

Research country of origin tends to follow nations that are active in track cycling competition. Whether publication of research directly explains track cycling success, or vice versa (i.e., reverse causality), cannot be shown with much certainty. However, it is notable that the four countries with the greatest contribution to the literature are frequently high performing in World Championships, World Series, Commonwealth Games, and Olympic Games. As per previous statements within this discussion, the published research is likely only a fraction of the true amount of research conducted within track cycling. Other highly competitive track cycling nations are very likely conducting major and minor projects aimed at understanding and improving performance, which are reserved for their own personal advantage.

Although research interest has been increasing, and at approximately the same rate as sport science research in general, the 71 publications included in this review demonstrate the relatively low volume of research specifically involving track cyclists. A quick search of PubMed reveals at least triple the number of results for ‘road cycling’ and ‘mountain-biking’ compared with ‘track cycling’. This discrepancy exists despite there being 21 Olympic and Paralympic track cycling events, compared with 15 events across road, mountain bike, and BMX events, combined. While transferability of knowledge exists across these disciplines, it is imperative that research addresses the needs of track cycling with more specificity to its demands.

### Limitations

A potential, albeit self-imposed, limitation of the present review was the decision to exclude studies with non-track cyclist populations (e.g., trained road cyclists) despite the possible relevance to parameters of track cycling (e.g., use of 4 km time trials as a performance measure). While these studies may be useful to consider alongside the track cycling-specific literature, as some of the included studies make clear, there are notable differences in the physiology and morphology of track and road cyclists. The purpose of this review was to map the literature to understand the scale and density of research and identify any areas potentially requiring attention specifically within track cycling. Therefore, given the scope of this research, we believe the exclusion of broader populations was reasonable.

The full-text articles of several records appearing in the screening process were not able to be located for appraisal. As such, some of these missing articles may have been excluded from the final review. In most cases however the available abstract provided sufficient information to discern eligibility for inclusion; appropriately categorise the study by domain, component, and scientific subdiscipline; and extract basic information about the study.

### Implications and Future Research

The mapped literature shows an apparent preference of researchers toward addressing the physical component of performance rather than tactical, technical, or mental components. Those studies also tend to be focused on the domains of testing and performance optimisation, with the training domain receiving considerably less attention. There is considerable overlap and interdependency between the domains of athlete preparation, therefore research conducted in any of the three likely has flow-on benefits for the others. Contrastingly, while there is interplay between the components of performance, and their effective integration contributes to success, they are more independent and therefore should be investigated as such to improve our understanding for application in track cycling. The limited research on tactical, technical, and mental components of performance may be limiting the ability of those within the sport to effectively improve athletes’ capabilities in those areas. The perspectives of coaches and athletes about specific research questions within each of these domains and components is invaluable to researchers, and therefore we should seek to gather their input from the earliest stages of study design through to dissemination. In particular, researchers must ask what is relevant and important to coaches and athletes for improving performance so that these can be addressed in a more targeted manner. Understanding the current needs of the sport and how the current literature aligns with those needs is critical to delivering a pipeline of research that is relevant and reliable in practice.

Furthermore, the dependence on quasi-experimental and especially non-experimental research may also limit the efficacy for implementation of research findings in evidence-based practices. These research designs also do not permit for understanding the causative effects of various interventions and can result in a greater risk of bias. We acknowledge the difficulties with administering well-controlled, randomised research in high-performance sport environments, but we do encourage researchers interested in track cycling, and the sport science community as a whole, to look for solutions to this issue so that we can improve the quality of our research and understanding of the demands and needs of the sport, and the true causative effects of our interventions and practices. The use of multicentre studies should be explored as one method for increasing the sampled populations available to researchers in answering valuable research questions. Closer collaboration with track cycling programmes and national sporting organisations, and their direct inclusion in research design and decision-making processes could be a possible solution to the research-quality dilemma.

## Supplementary Information

Below is the link to the electronic supplementary material.

Supplementary file1 (PDF 127 kb)

## Declarations

### Availability of data and material

This review was registered on OSF and all search results, data, interactive visualisations, and registration and protocol information are available online at http://osf.io/wt7eq.
